# Structure-Functional Examination of Cysteine Synthase A (CysK) from *Limosilactobacillus reuteri* LR1

**DOI:** 10.3390/ijms27010327

**Published:** 2025-12-28

**Authors:** Anastasia A. Pometun, Evgenii K. Les, Alla V. Chernobrovkina, Anastasiia V. Gorbovskaia, Natalia Yu Chikurova, Anastasia A. Loginova, Alexey N. Antipov, Nadezhda N. Mordkovich, Leonid A. Shaposhnikov, Svyatoslav S. Savin, Sergey Yu Kleymenov, Ilya O. Matyuta, Konstantin M. Boyko, Mikhail E. Minyaev, Dmitry M. Hushpulian, Evgenii V. Pometun, Vladimir I. Tishkov

**Affiliations:** 1 Bach Institute of Biochemistry, Research Center of Biotechnology of the Russian Academy of Sciences, 33, bld. 2 Leninsky Avenue, 119071 Moscow, Russia; evglesk2001@gmail.com (E.K.L.); anastasiia.gorbovskaia@chemistry.msu.ru (A.V.G.); chikurova.nu@yandex.ru (N.Y.C.); loginovaa.anast@gmail.com (A.A.L.); alex131313@yandex.ru (A.N.A.); serkovan@mail.ru (N.N.M.); shaposhnikovleo@gmail.com (L.A.S.); savinslava@gmail.com (S.S.S.); s.yu.kleymenov@gmail.com (S.Y.K.); i.matyuta@fbras.ru (I.O.M.); kmb@inbi.ras.ru (K.M.B.); hushpulian@gmail.com (D.M.H.); tishkovvi@my.msu.ru (V.I.T.); 2 Institute of Medicine, Peoples’ Friendship, University of Russia Named After Patrice Lumumba, Miklouho-Maklaya, 8, 117198 Moscow, Russia; 3 Department of Chemistry, Lomonosov Moscow State University, Leninskie Gory, 1-3, 119991 Moscow, Russia; chernobrovkina@analyt.chem.msu.ru; 4 Koltzov Institute of Developmental Biology of Russian Academy of Sciences, Vavilova, 26, 119334 Moscow, Russia; 5 N. D. Zelinsky Institute of Organic Chemistry, Russian Academy of Sciences, Leninsky Avenue, 47, 119991 Moscow, Russia; mminyaev@mail.ru; 6 Federal State Autonomous Educational Institution of Higher Education, I.M. Sechenov First Moscow State Medical University (Sechenovskiy University), Trubetskaya St., 8, Building 2, 119048 Moscow, Russia; epometun@gmail.com

**Keywords:** cysteine synthase A, lactobacilli, nosocomial infections, enzymatic activity, crystal structure, model structure studies

## Abstract

This study presents a comprehensive analysis of cysteine synthase A (CysK) from *Limosilactobacillus reuteri* LR1 (LreCysK), an enzyme involved in the biosynthesis of L-cysteine. This protein supports crucial cellular functions such as sulfur metabolism, antioxidant defense, detoxification, and protein synthesis. Previously, the gene encoding LreCysK was cloned, and the enzyme with His-tag on the N-terminus was obtained in active and soluble form. Here, kinetic parameters of the enzyme were determined by the previously developed high-pressure liquid chromatography (HPLC) and ninhydrin methods. It was found that LreCysK has similar K_M_^OAS^ and *k_cat_* as CysKs from *Escherichia coli* and from the model plant *Arabidopsis thaliana*. The thermal stability of LreCysK was studied using differential scanning calorimetry. It was revealed that the melting point of the enzyme increases to almost 90°C when Pyridoxal-5 phosphate (PLP) is added, indicating that the stability of the enzyme complex with PLP is relatively high. Structural studies revealed that LreCysK is a dimer, and its active site is similar to those of other enzymes, but exhibits some features characteristic of lactobacilli CysKs (GISA), as well as unique residues, such as Ile50. Also, the potential biotechnological applications of LreCysK are discussed. These findings enhance our understanding of LreCysK’s biochemical versatility and its potential applications in biotechnology and medicine.

## 1. Introduction

Cysteine synthase A (CysK, EC 2.5.1.47) is a pyridoxal phosphate (PLP)-dependent enzyme that belongs to the transferase class and catalyzes the formation of L-cysteine from sulfide and O-acetyl-L-serine (OAS). This reaction represents a critical step in the sulfur assimilation pathway. This has a vital role in maintaining sulfur homeostasis and supporting cellular functions such as antioxidant defense, detoxification, and protein synthesis [1]. The significance of cysteine biosynthesis extends beyond individual metabolic pathways; it is a cornerstone in the synthesis of glutathione, an essential antioxidant that protects cells from oxidative stress and maintains redox balance. In the context of bacterial pathogens, cysteine biosynthesis can confer a survival advantage, particularly under conditions of oxidative stress or nutrient limitation. Therefore, CysK inhibitors are being actively studied as potential antimicrobial agents [2,3]. Additionally, CysK plays a role in contact-dependent growth inhibition, L-isoleucine production, and biofilm formation, which may suggest different possible applications of this enzyme [3,4].

*L. reuteri* is the probiotic strain, and its properties are well studied and include the control of insulin sensitivity, anti-inflammatory effects, synthesis of vitamins, as well as low molecular weight compounds with antibacterial effect [5,6]. The study of CysK from *L. reuteri* (LreCysK) is of particular interest due to its potential role in conferring resistance to environmental stressors and enhancing the probiotic effects of the bacterium, such as modulating the host immune response and inhibiting pathogenic microorganisms [2,7]. Understanding the biochemical properties of LreCysK, including its kinetics, thermostability, and structure, could provide valuable insights into its role in *L. reuteri*’s probiotic functions, especially in its interaction with pathogenic bacteria and potential applications in biotechnology and medicine. Due to its crucial physiological role in cysteine synthesis in various pathogenic bacteria, specific inhibitors of CysK can exhibit an antibacterial effect [3,4]. *L. reuteri* is a component of normal gut microbiota. Consequently, structural studies of CysK from this bacterium may help in developing specific inhibitors of this enzyme from pathogenic bacteria and in preventing the inhibition of normal microbiota growth.

One of the most challenging problems in modern medicine is the emergence of pathogenic bacteria with multiple drug resistance. That is why many researchers try to find new sources of antibacterial substances. Recent studies have shown that *L. reuteri* LR1 can inhibit the growth of *Klebsiella pneumoniae*, a pathogenic bacterium known for its multidrug resistance [8]. Exoproteome analysis demonstrated that *L. reuteri* responds to *K. pneumoniae* by secreting new enzymes of various groups, including peptidases, nucleosidases, and metabolic enzymes. One of the proteins found was metabolic enzyme cysteine synthase A (CysK), which may contribute to its antagonistic effect [8]. Additionally, recent research showed the possible presence of such activity [9]. This discovery suggests a potential mechanism through which *L. reuteri* could exert a competitive advantage over pathogenic bacteria by modulating its metabolic pathways. Since *K. pneumoniae* is a pathogenic organism that causes sepsis, pneumonia, inflammation of the urinary system, and problems with the liver and kidneys, new approaches to combat it are needed, especially since resistance to classic antibiotics is increasing every year [10].

The literature contains data on the structural organization of CysK from several bacteria (6Z4N, 6Z4N, 2Q3C, 5XOQ, 4I1Y, 2EGU) and protozoa (4JBN, 3SPX, 8B9Y). It is known that for most structures, CysK is a dimer consisting of two domains, located closer to the N- and C-termini of each subunit. There is extremely little data on the structure of CysK from lactobacilli in the scientific literature. PLP binds to the amino acid residue of lysine by a covalent bond similar to other PLP-dependent enzymes to form a Schiff base [11]. Previously, the gene encoding cysteine synthase A from *L. reuteri* was cloned, and we obtained the enzyme in an active and soluble form [12]. Additionally, a method for determining activity using Hydrophilic Interaction Liquid Chromatography (HILIC) chromatography was developed [12]. In this paper, we present data on the biochemical and structural characteristics of this enzyme.

## 2. Results and Discussion

### 2.1. Obtaining the LreCysK Enzyme with His–Tag

CysK with a His–tag is expressed in *E. coli* cells in a soluble form, with a yield of approximately 40 mg per liter of nutrient medium. The degree of purity of the enzyme was confirmed using SDS–PAGE (Supplementary Figure S1). Tandem MALDI-TOF/TOF mass spectroscopy confirmed the sequence of LreCysK. The percentage after MALDI analysis, using the theoretical sequence, was 92% (Supplementary Figure S2). The enzyme was obtained in a highly purified and soluble state with a degree of purity of at least 95%. The theoretical mass of the LreCysK monomer with His–tag is 33.12 kDa, which is also consistent with the SDS–PAGE.

### 2.2. Determination of the Oligomeric Composition of the Enzyme

**Figure 1:**
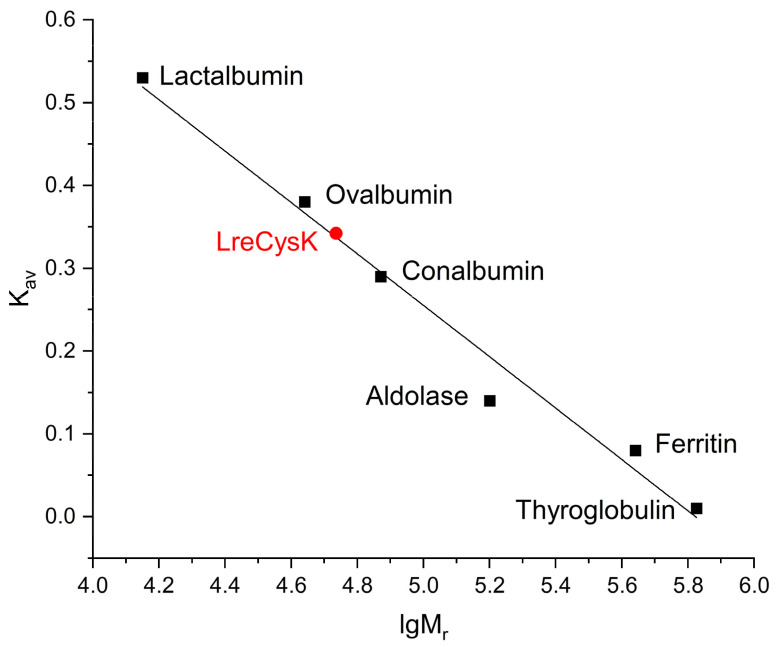
The calibration curve of analytical gel filtration. LreCysK enzyme is shown in red.

The oligomeric composition of CysK was determined using analytical gel filtration. The calibration curve with LreCysK plotted on it is presented in Figure 1.

The theoretical monomer mass of LreCysK with His-tag at N-terminus, calculated using the amino acid sequence, is 33.12 kDa. Parameter K_av_ was calculated using the formula K_av_ = (V_e_ − V_O_)/(V_C_ − V_O_), where V_O_ is the column void volume, V_e_ is the elution volume, and V_C_ is the geometric column volume. For all proteins used for the calibration curve, the parameter K_av_ was calculated (Table S1, Supplementary Figure S3). The M_r_ for CysK was calculated from the dependence of K_av_ on lgM_r_.

The molecular mass of LreCysK, as determined by gel filtration, was 54 kDa, corresponding to a dimer. This correlates with the structures of different CysK enzymes, which are typically dimers.

### 2.3. Kinetic Parameters of LreCysK

The widely used method for studying CysK kinetics is the spectrophotometric method, which utilizes the ninhydrin acidic reagent (NAR). In this method, cysteine binds with ninhydrin to form a pink product, measured at 560 nm. This method has several disadvantages, including low stability of the colored reaction product (color saturation decreases over time), limited specificity for cysteine (ninhydrin can interact with other amino acids), and a relatively high detection limit (approximately 0.5–1 mM). In this study, we employed the ninhydrin method and developed a novel approach using HILIC for the separation of cysteine from other components of the mixture and its subsequent determination [12]. Using HPLC generally provides better results for this task due to its ability to separate the target analyte from other substances. HILIC is a relatively new method for measuring enzymatic activity, providing better and faster separation of polar substances. To separate a mixture of complex composition: two substrates (OAS and sulfide), a potential product (cysteine), and components of a buffer solution (HEPES), the following composition of the mobile phase was chosen: 12 mM phosphate buffer, pH 6.5 (aqueous) and acetonitrile (22:78 vol.%) according to [12]. It is essential to be able to separate cysteine and HEPES, as well as to separate them from the two substrates.

The kinetic parameters are presented in Table 1. This table also shows the kinetic parameters of other CysK enzymes. Parameters for the enzymes obtained in this work are in bold. Supplementary Figure S4 shows the dependence of the enzymatic reaction rate on the substrate concentration used to calculate the kinetic parameters for the LreCysK using HILIC and ninhydrin methods.

Results demonstrate that both methods yield similar K_M_ values for OAS; however, the *k_cat_* measured by the ninhydrin method is 2.5 times higher than that measured by our new method. This may be due to the nonselective reaction between NAR and other reaction components, except cysteine. When comparing the kinetic parameters of the LreCysK enzyme obtained in this work with those from other sources, it is notable that the Michaelis constant for OAS is typically 10 to 100 times higher than that for sulfide. The only exception is the enzyme from *L. casei* FAM18110. Interestingly, although both enzymes are derived from lactobacteria, their characteristics differ. LreCysK is closer to CysK from *E. coli*, *S. typhimorium,* and *A. thaliana*, which allows us to assume their common evolutionary origin. AthCysK is indeed evolutionarily close to LreCysK (Supplementary Figure S5), but EcoCysK is an evolutionarily distant enzyme. The similarity in the kinetic parameters of EcoCysK and LreCysK can be attributed to their shared habitat—the human gastrointestinal tract.

### 2.4. Temperature Stability of LreCysK

**Figure 2:**
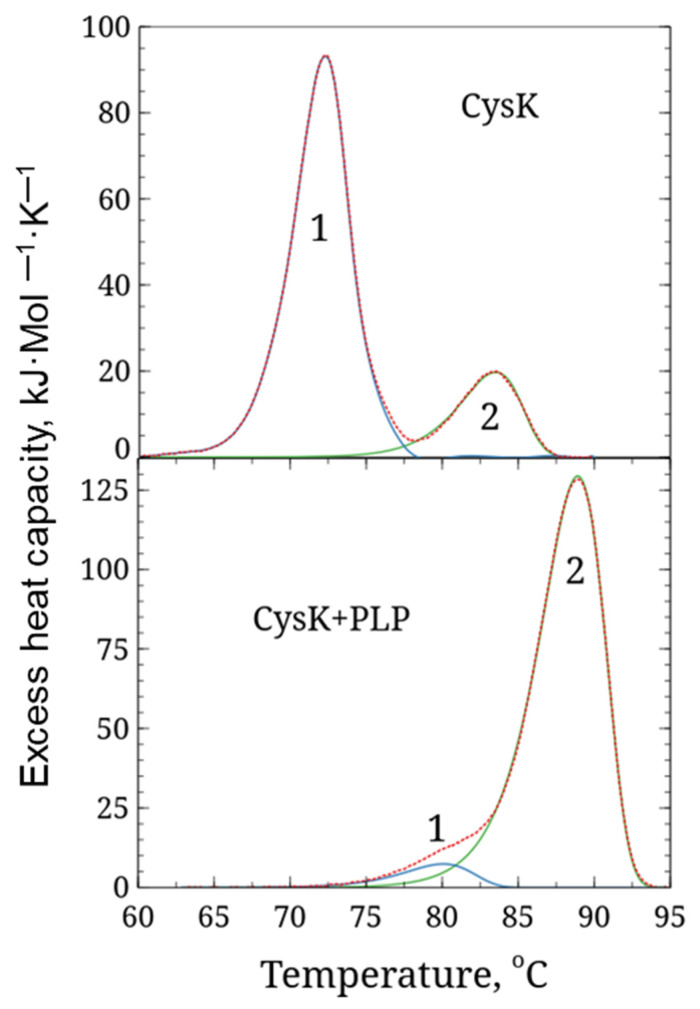
DSC curves for LreCysK apo and holo forms. 1, 2—separate domains. CysK (**upper graph**)—enzyme incubating without additional PLP, CysK + PLP (**lower graph**)—enzyme incubating with additional PLP. Red dotted lines—before deconvolution, and solid line—after deconvolution of domains (blue and green line correspond to the first and second domain respectively).

Differential scanning calorimetry (DSC) was used to study the temperature stability of LreCysK. During the experiment, the temperature of the sample cell and the comparison cell was linearly increased, and the change in heat capacity was monitored. Since protein denaturation is a phase transition, the experimental curve shows an increase in the change in heat capacity of the sample with a peak at the temperature at which the maximum rate of denaturation occurs, and which is used as a characteristic of the protein’s thermal stability (the so-called phase transition temperature T*_m_*). DSC data for both variants of LreCysK are presented in Figure 2 and Table 2.

DSC analysis revealed two well-distinguishable calorimetric domains with melting points of 72.3 °C and 83.5 °C in the apo form of CysK, as well as two poorly distinguishable calorimetric domains in the holo form (in the presence of PLP) with melting points of 80.1 and 88.9 °C, respectively. A cardinal shift in melting point and redistribution of denaturation enthalpy between domains indicated that the holo form of CysK is more stable than the apo form. This effect is often observed in PLP-dependent enzymes and has been demonstrated for serine dehydratase, ornithine δ-aminotransferase, and tyrosine aminotransferase [22,23]. The observed thermal stability of LreCysK is likely due to a combination of factors, including a highly hydrophobic core, extensive hydrogen bonding, and ionic interactions. The presence of proline residues in strategic locations could also contribute to the enzyme’s rigidity and resistance to thermal denaturation. Further studies involving mutagenesis could help identify specific residues or regions responsible for this thermostability, providing insights for engineering more stable enzyme variants. The high thermal stability observed is a desirable trait for industrial applications, where enzymes are often exposed to high temperatures.

### 2.5. Structural Analysis of LreCysK

**Figure 3:**
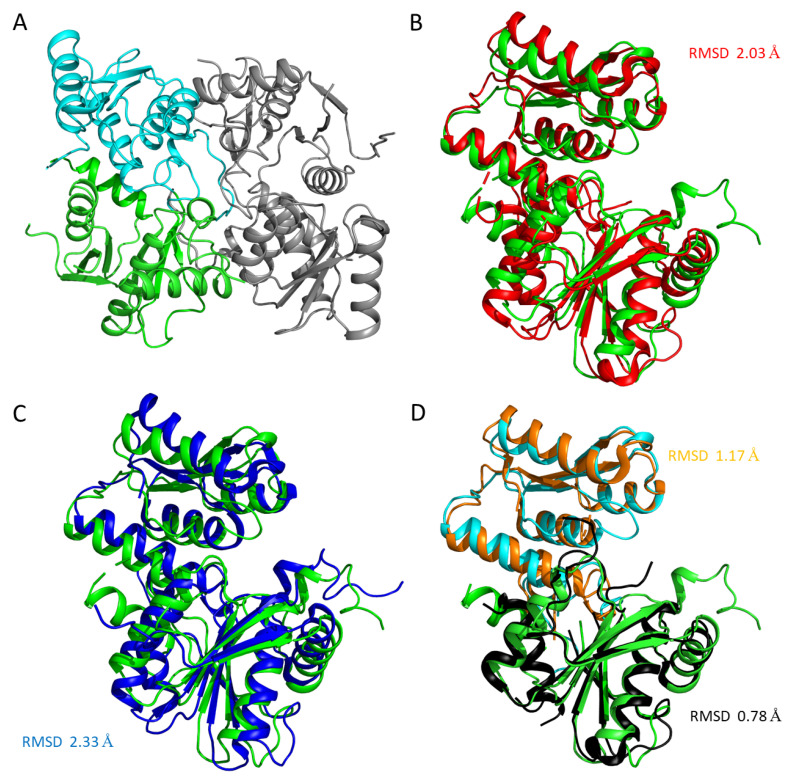
(**A**) The LreCysK*apo* functional dimer. The large domain in one subunit is shown in green, the small domain—cyan, and the adjacent subunit—gray. Superposition of the subunits of LreCysK*apo* (green) with MulCysK*apo* (red, (**B**)) and GkaCysK*apo* (blue, (**C**)). (**D**) Superposition of large and small domains of LreCysK*apo* (green and cyan for LreCysK large and small domains respectively) and MulCysK*apo* (black and orange for MulCysK large and small domains, respectively). Panels (**B**)–(**D**) are presented in the same orientation.

The crystal structure of the apoenzyme (LreCysK*apo*) was solved at a resolution of 2.2 Å. The asymmetric unit contained two subunits related by 2-fold non-crystallographic symmetry (NCS). (RMSD between Cα atoms between subunits is 0.3 Å) organized in a dimer whose buried area comprised 16% of the total surface area of each subunit (Figure 3). According to gel filtration, LreCysK is also a dimer in solution. The subunit of LreCysK*apo* consists of large (residues 21–43 and 161–313) and small (residues 1–20 and 44–152) domains, similar to those of other CysK proteins described in [24,25]. We assume that these domains are the N- and C-terminus domains, which often occur in the structure of cysteine synthases [26]. The full structure is also typical for cysteine synthases: every domain consists of α/β-folds with the central β-sheet surrounded by α-spirals [27]. The closest structural homologs of the LreCysK*apo* are cysteine synthase from *Mycobacterium ulcerans* (MulCysK*apo*, PDB ID 4I1Y, RMSD between Cα atoms of subunits is 2.03 Å^2^, Table 3) and O-acetylserine sulfhydrylase from *Geobacillus kaustophilus* HTA426 (GkaCysK*apo*, PDB ID 2EGU, RMSD is 2.33 Å). Structure superposition (Figure 3B, C) and RMSD showed severe differences between LreCysK*apo* and MulCysK*apo,* with GkaCysK*apo,* but the RMSD of Cα atoms between subunits of MulCysK*apo* and GkaCysK*apo* is 1.3 Å (95% of the aligned residues). To elucidate the reasons for this difference, subunits were superimposed by domains. RMSD between large and small domains of LreCysK*apo* and MulCysK*apo* are 0.78 Å (97% of aligned residues) and 1.17 Å (98% of aligned residues), respectively (Figure 3D), indicating that the large and small domains of LreCysK*apo* are similar to those from MulCysK*apo*. The high RMSD between subunits was caused by differences in the relative orientation of the large and small domains in LreCysK*apo* compared to the reference structures.

**Figure 4:**
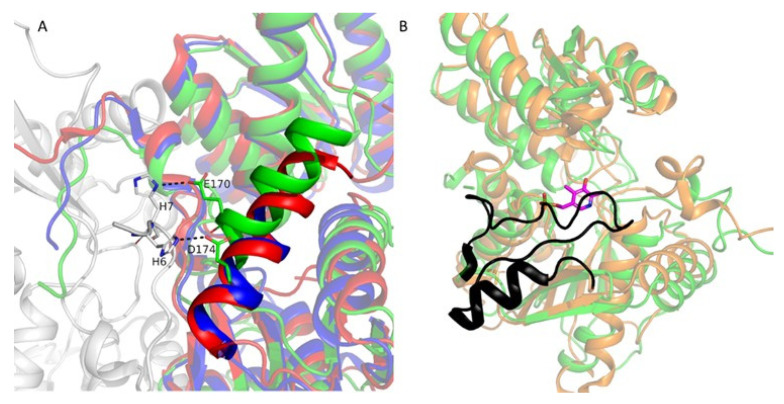
(**A**) Superposition of LreCysK*apo* (colored by subunits: green and gray), MulCysK*apo* (red), and GkaCysK*apo* (blue). Fitted regions are shown semi-transparent for clarity. Only the residues described in the text are shown. Polar contacts are shown as black dotted lines. (**B**) Superposition of LreCysK*apo* and StyCysK*holo*. LreCysK*apo* is shown in green, StyCysK*holo* is shown in orange, residues 206–240 in SenCysK*holo* are shown in black, and PLP is shown in magenta sticks.

Moreover, *α*-helix (residues 162–172) (Figure 4) in LreCysK*apo* has a different conformation than in MulCysK*apo* (residues 152–166) and GkaCysK*apo* (residues 157–166) (Figure 4). In LreCysK*apo,* residues of this *α*-helix (E170 and D174) form polar contacts with N-terminus His–tag residues (H6 and H7) from the adjacent subunit (Figure 4A). The addition of His–tag to the N-terminus of the enzyme could change the conformation of this *α*-helix. We assume that these changes don’t influence the catalytic activity since this *α*-helix is not part of the active site and presumably only stabilizes the conformation. Residues 214–240 and 213–235 in subunits A and B, respectively, have no electron density. Moreover, this region is absent in MulCysK*apo* (residues 183–204) and GkaCysK*apo* (residues 207–229). This region has no electron density in all apo form structures due to the high mobility. In StyCysK holo form structure (PDB ID 6Z4N, StyCysK*holo*), this region (residues 206–240, black in Figure 4B) is clearly visible and shields the active site from the solvent and fixes the PLP in the active site.

Table 3 shows the comparison of the LreCysK*apo* structure with the known crystal structures of CysK from other organisms. All mentioned structures have one molecule per asymmetric unit. We can indicate that, except for GkaCysK and AthCysK, practically all the mentioned structures are dimers.

**Figure 5:**
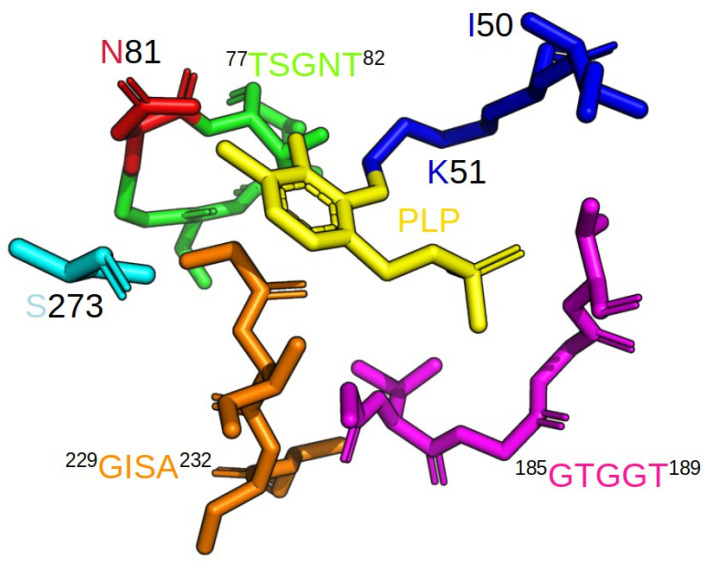
Different fragments of the polypeptide chain of LreCysK*apo* with artificially added PLP are shown in the following: blue—dipeptide ^50^IK^51^, green—loop ^77^TSGNT^82^, red—residue Asn81 in loop ^77^TSGNT^82^, magenta—loop ^185^GTGGT^189^, orange—loop ^229^GISA^232^, Cyan—residue Ser273.

**Figure 6:**
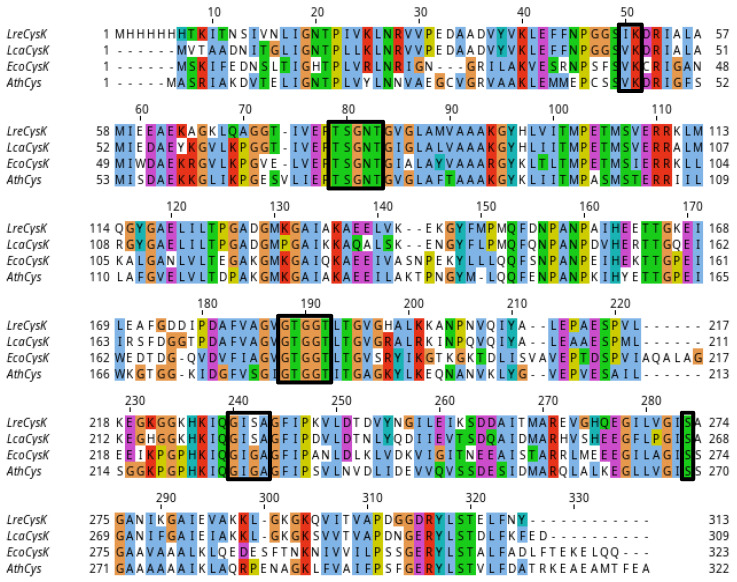
Multiple sequence alignment of well-studied CysK from several organisms. LreCysK—CysK from *L. reuteri* (GenBank: MBU5982312.1)*,* the enzyme of interest in this paper, LcaCysK—CysK from *L. casei* (GenBank: ADR71221.1), EcoCysK—CysK from *E. coli* (GenBank: CAA0274471.1), AthCysK—CysK from *A. thaliana* (NCBI Reference Sequence: NP_001190732.1). Main loops and amino acid residues of the active site, such as ^77^TSGNT^82^, ^185^GTGGT^189^, ^229^GISA^232^, Ser273, and the binding site of PLP, ^50^IK^51^, are highlighted in boxes.

Since the crystal structure of our LreCysK*apo* protein contained two unresolved loops (positions 153–160 and 214–240), we modelled these loops. Our further structural investigations of LreCysK*apo* were made on this model structure. In Figure 5, the active site of LreCysK*apo*, featuring highly conserved motifs, is presented based on this model structure. Analysis of amino acid sequences of well-studied CysKs was already done by us and published in [28]. In this paper, we focused on certain sequences of enzymes, closely related to LreCysK, based on their biochemical characteristics (Table 2) and phylogenetic analysis (Figure S5). The alignment of the similar CysKs based on these factors is presented in Figure 6.

The conserved lysine residue (Lys51 in LreCysK*apo*) plays a pivotal role in forming a Schiff base with the PLP cofactor, a highly conserved feature among PLP-dependent enzymes. This interaction is crucial for catalytic activity, as it facilitates the nucleophilic attack on the OAS substrate. The pyrimidine ring of PLP fits the small hydrophobic cavity of the active site and is usually stabilized by a Val residue, although in the case of LreCysK, Ile50 plays this role. Glycine-rich region ^185^GTGGT^189^ creates a loop near the phosphate group of the PLP, making hydrogen bonds with the oxygen atoms. The deprotonated 3′-OH group of the PLP creates a hydrogen bond with the amide nitrogen of the Asn81, and the nitrogen of the pyrimidine ring of PLP creates a hydrogen bond with the Ser273, thus being the last point of the coenzyme stabilization. The catalytic site in the case of LreCysK consists of amide atoms of a highly conserved region ^77^TSGNT^82^, which correlates with sequences of practically all known CysK structures. The other conserved region crucial for catalysis is ^229^GISA^232^ (which is usually GIGA, as can be seen from other structures). We assumed that GISA is a structural characteristic of lactobacilli CysKs as both LreCysK and LcaCysK possess it. This assumption is supported by research on amino acid sequences from different lactobacilli. Most of them have the GISA, not the GIGA (Supplementary Figure S6). During catalysis, when the intermediate product is formed, these two loop regions close the active site, limiting solvent access to the highly hydrophobic region of the active site and thereby protecting the intermediate product.

Amino acid alignment of LreCysK with several other CysK enzymes showed a high number of aligned regions with over 90% of identity (Table 4, Figure 6). For alignment, we selected the enzyme from another *Lactobacillus* species (*L. casei*) and two other enzymes that share similar kinetic properties with LreCysK (Table 2). Across the alignment, several highly conserved regions are observed, particularly in key functional motifs related to substrate binding and catalysis as discussed earlier. These regions are represented by loops that have identical or similar residues across all organisms, indicating their importance in maintaining CysKs enzymatic function. Curiously, the two lactobacterial enzymes LreCysK and LcaCysK (the latter being from *L. casei* FAM18110) have a difference in one catalytically important region. This region is ^347^GISA^350^ (numbered as in alignment), and it closes the catalytic cavity during the reaction as discussed earlier. Other CysK enzymes have GIGA in this region instead. Apparently, the substitution of glycine for serine in the GISA loop, characteristic of lactobacilli, does not significantly affect the Michaelis constant for o-acetylserine. Since the K_M_^OAS^ in LreCysK is similar to the corresponding values for CysK from *A. thaliana* and *E. coli,* which have GIGA in this region. This allows us to assume that LcaCysK has some other important amino acid residues that affect the binding of o-acetylserine.

## 3. Materials and Methods

### 3.1. Obtaining LreCysK and Protein Expression

The gene encoding CysK enzyme was cloned previously from the *L. reuteri* LR1 bacteria (the strain was kindly provided by the All–Russian Dairy Research Institute (VNIMI)) with the addition of a fragment encoding a six-histidine residue sequence (His–tag) to the N-terminus of the enzyme for acceleration and simplifying the enzyme purification process [12].

Sequencing results showed that the genetic construct contains only the CysK gene with the target insert (His–tag at the N-terminus of the enzyme). The size of the CysK gene with a His–tag was 939 bp, the gene encodes a protein with a length of 313 amino acid residues and a size of 33.12 kDa, the accession number of the CysK enzyme from *L. reuteri* without His–tag is MBU5982312.1.

The plasmid, containing the gene encoding LreCysK, was used to transform *E. coli* BL21(DE3) CodonPlus/pLysS cells resistant to chloramphenicol. Expression of the enzyme was performed according to the algorithm from previous work [29]. The resulting cell suspensions were stored at –20 °C until purification.

### 3.2. Enzyme Purification

*E. coli* cells containing the CysK enzyme were lysed on an ultrasonic disintegrator. The cell debris was settled on an Eppendorf 5804 R centrifuge at 4 °C and 5000 rpm, and the supernatant was transferred into clean plastic tubes with a volume of 50 mL. Purification was carried out on an AKTA Start chromatography system using a HisTrap FF 1 mL column (Cytiva, Marlborough, MA, USA). The column was pre-equilibrated with buffer A (0.05 M Tris–HCl, 0.5 M NaCl, 0.02 M imidazole, pH 7.5). The supernatant after cell disruption in buffer A was applied to the column at a flow of 0.5 mL min*^−^*^1^. After the disappearance of the absorption peak at 280 nm of impurity proteins that did not bind to the column, the target enzyme was eluted with a linear gradient of increasing concentration of buffer B (0.05 M Tris–HCl, 0.5 M NaCl, 0.5 M imidazole, pH 7.5). The resulting enzyme solution was desalted using size-exclusion chromatography on a Sephadex G25 (Cytiva, Marlborough, MA, USA) column into a solution containing 0.05 M Tris-HCl, pH 7.5. The purity of the resulting enzyme solution was confirmed by SDS–PAGE as described in [30].

Confirmation of the amino acid sequence of the CysK was performed using MALDI-TOF/TOF mass spectrometry as described previously [29].

### 3.3. Determination of Oligomeric Composition of the Enzyme by Size-Exclusion Chromatography

The oligomeric composition of the enzyme was determined using analytical size-exclusion chromatography (gel filtration) on an AKTA Start chromatograph on a HiLoad 16/600 Superdex 200 pg column (Cytiva, Marlborough, MA, USA). To construct a calibration curve, a set of molecular weight standards for gel filtration and analytical electrophoresis, Gel Filtration Calibration Kit HMW (Cytiva, Marlborough, MA, USA) was used. Column equilibration and calibration were performed according to the manufacturer’s protocol. The test sample was added to the column in an amount of 1 mg, and its retention time on the column was observed. Next, using a previously constructed calibration curve, the molecular mass of the sample was determined and compared with the theoretical mass of the monomer, from which a conclusion was drawn about the oligomeric composition of the enzyme.

### 3.4. Carrying out an Enzymatic Reaction

The enzymatic reaction was carried out using 99% pure O-acetyl-L-serine (OAS), sodium sulfide nonahydrate from Sigma–Aldrich (St. Louis, MO, USA), PLP monohydrate from Loba-Chemie Austranal-Praparate (Fischamend, Austria), and water purified by a MilliQ unit. The reaction was carried out as follows: the desired stock concentrations of PLP, OAS and sulfide were prepared by weighing and dissolving reagents in 10 mM HEPES buffer pH 7.5, then both substrates were added at the required concentrations (diluted with HEPES buffer when needed) to plastic test tubes (total volume 1.5 mL) to the 1000 µL volume, PLP was added to the final concentration 5 µM. For each concentration of substrate, three replicates were made. Next, 10 µL of purified CysK solution at a concentration of approximately 290 µg mL*^−^*^1^ (for HILIC) and 77 µg mL*^−^*^1^ (for ninhydrin method) was added to each sample (the final concentration in the solution is 2,9 (for HILIC) and 0.77 (for ninhydrin method) µg mL*^−^*^1^) and stirred. At certain time intervals, the enzymatic reaction was stopped by adding 10 µL of concentrated HCl at different times of the reaction for conducting analysis with HILIC, as previously developed [9], or the ninhydrin method. The prepared samples were then analyzed. For measuring the kinetics of the enzyme, one substrate was added at a fixed, saturating concentration to the mixture, while the concentration of the other was varied.

### 3.5. Conducting the Analysis Using Nynhidrin Reaction

For analysis, 300 µL of modified Ninhydrin Acid Reagent (NAR, comprising 250 mg of ninhydrin, 11 mL of concentrated acetic acid, and 4 mL of concentrated HCl) was added to the reaction mixture, resulting in a final volume of 1.02 mL. The mixtures were heated to 95 °C for 10 minutes. After this, mixtures were cooled on ice for 1–2 min, and 650 µL of ethanol was added to them. The presence of cysteine in mixtures was gained by absorption measurement at a wavelength of 560 nm. Cysteine concentrations were calculated using a calibration curve that was linear in the range of 0.1 mM to 0.7 mM.

### 3.6. Conducting the Analysis Using HILIC

For analysis, 99% pure cysteine from Sigma–Aldrich (St. Louis, MO, USA), water purified using a MilliQ unit, and HPLC gradient grade acetonitrile (Panreac, Darmstadt, Germany) were used. The experiments were conducted using a Dionex UltiMate 3000 Liquid Chromatography system (Dionex, a part of Thermo Scientific, Sunnyvale, CA, USA), equipped with a gradient pump, autosampler, column thermostat, and diode array detector. Chromatograms were recorded using a personal computer and the Chromeleon 7.0 software package (Thermo Fisher Scientific, Waltham, MA, USA). A 100 × 3.0 mm i.d. stainless steel column was packed with a hydrophilic stationary phase based on 3-aminopropylsilica with amide groups in a functional layer, which was prepared via multicomponent Ugi reaction according to [12,31,32] by means of 2-acetylpyrrole, 2-morpholinoethyl isocyanide, and glycolic acid. This column exhibited high selectivity towards amino acids [31], and a column with a similar functional layer [32] demonstrated high stability during enzymatic activity determination. The separation and analysis were carried out as described in [12]. A set of “blank” injections of a substrate mixture solution in HEPES and HEPES buffer solution alone was also conducted to check for corresponding impurities. Kinetic parameters of the reaction were determined using the recently described HILIC method, and calculations were carried out in Origin 2018.

### 3.7. Kinetic Parameters Determination for LreCysK

The dependence of the enzymatic reaction rate on the substrate concentration was obtained as described above. This dependence was analyzed using Origin Pro 8.5. The Michaelis constant K*_M_* was determined using nonlinear regression in the range of substrate concentrations of 0.5 K*_M_*–5 K*_M_*. From the same dependence, the maximum speed of the enzymatic reaction, V*_max_*, was determined. The concentration of the purified enzyme was assayed by measurement of solution absorption at 280 nm (A^280^) and calculated using the equation C_protein_ = A^280^/(ε_protein_ × l), where ε_protein_ was 11,920 M^−1^·cm^−1^. The A^280^ value was corrected by absorption of PLP at the same wavelength. Using V*_max_* and C*_protein_*, the rate constant of the enzymatic reaction, *k_cat_*, was calculated from the equation V*_max_* = *k_cat_* × C*_protein_*.

### 3.8. Study of Enzyme Thermostability

To study the thermal stability of the LreCysK enzyme, differential scanning calorimetry (DSC) was used. DSC was carried out using a MicroCal VP-Capillary differential scanning microcalorimeter (Malvern Instruments, Northampton, MA, USA) equipped with 140 µL tantalum capillary cells. The experimental protocol involved a heating rate of 1 °C·min^−1^, with an overpressure maintained at 3 bars to prevent boiling inside the cells. Enzyme samples were prepared at concentrations ranging from 1 to 2 mg·mL^-1^ in 0.1 M Na-phosphate buffer (pH 7.0). For samples containing PLP, the cofactor was added at a concentration of 1.0 mM. The instrumental baseline was established by recording buffer-to-buffer scans, which were subsequently subtracted from the protein data to correct for background signals. The resulting calorimetric peaks corresponding to individual domains were deconvoluted using the Zubov-Markov method [33], implemented through an original procedure developed for the MATLAB R2025b computing environment (MathWorks, Natick, MA, USA).

### 3.9. Multiple Sequence Alignment (MSA)

For MSA, Clustal-Omega was selected using the program Clustalo (version 1.2.4) https://www.ebi.ac.uk/jdispatcher/msa/clustalo (accessed on 7 September 2025). Visualization of MSA was performed using the Jalview 2.11.5.0 program. For visualization, Clustal colors were chosen in Jalview 2.11.5.0. [software] https://www.jalview.org/.

### 3.10. Crystallization and X-Ray Diffraction Data Collection

Initial crystallization screening was done by the “sitting drop” vapor diffusion method on a robotic system (Oryx4, East Garston, Berkshire, UK) with 96-well VDX plates (Hampton Research, Aliso Viejo, CA, USA) and commercial crystallization screens from Hampton Research (Aliso Viejo, CA, USA) and Molecular Dimensions Inc. (Holland, OH, USA). The N-terminal His-tagged LreCysK apo form was concentrated to 15 mg·mL^-1^ in 0.05 M Tris-HCl buffer, pH 7,5. The protein and crystallization solution were mixed in the ratios 2:1, 1:2 (using a 0.3 µL drop volume), and 1:1 (using a 0.2 µL drop volume). The reservoir contained 50 µL of the precipitant solution. The initial crystallization hit was observed under the following conditions: 0.15 M Lithium sulfate monohydrate, 0.1 M Citric acid, pH 3.5, 18% PEG6000 at all ratios at 288 K. During the optimization (done using “hanging drop” vapor-diffusion method in 24-well VDX plates (Hampton Research) the drop and the precipitant solution volumes were increased by 10 times.

Crystals of the LreCysK were briefly soaked in a mother liquor containing 20% ethylene glycol as a cryoprotectant, and subsequently flash-frozen in liquid nitrogen. The dataset was collected at 100 K at Rigaku OD XtaLAB Synergy-S (IOC RAS, Moscow, Russia). The datasets for the enzyme were indexed, integrated, and scaled using the CrysAlisPro software CrysAlisPro 1.0.43 (Oxford Diffraction/Agilent Technologies UK Ltd., Yarnton, UK). Space group was suggested by Pointless [34] as P2_1_ (Table 4).

The structure of LreCysK*apo* was obtained using the MOLREP program [35] by molecular replacement with the atomic coordinates of CysK from *Planctomyces limnophilus* (PDB ID: 5XOQ) as the starting model. The REFMAC5 program of the CCP4 suite (Harwell Science and Innovation Campus, Didcot, UK) [36] was used for the refinement with isotropic B-factors. The model rebuilding was carried out using the COOT interactive graphics program [37].

In the final model, an asymmetric unit contained two copies of the protein (555 visible residues), 350 water molecules, two PEG molecules, 6 ethylene glycol molecules, and 4 SO_4_^2−^ ions from the crystallization solution. 1–4 N-terminal, 152–161 and 213–241 residues in subunit A and 1–5 N-terminal, 155–160 and 212–236 residues in subunit B have no electron density.

### 3.11. Structure Analysis

The visual inspection was performed with the PyMOL Molecular Graphics System, Version 4.6 (Schrödinger, New York, NY, USA) and the COOT program. Structures were compared and superposed with the PDBeFOLD 2.58 (EMBL-EBI, Hinxton, UK) program [38]. The contact analysis was done using PDBePISA 1.48 (EMBL-EBI, Hinxton, UK) [39].

### 3.12. Structure Modelling

The model of LreCysK, which contains all missing residues in the crystal structure, was generated using the following procedure. Missing loops were created manually with the Discovery Studio 3.0 software suite (Accelrys, San Diego, CA, USA) using the insertion tool of residues according to the protein sequence received by MALDI-TOF/TOF for correct visualization of the active site, because we can’t see GISA, the active site’s one of the main loops, in our 3D structure. The inserted loop was refined with the “Loop refinement” protocol as implemented in Discovery Studio. Missing residues according to the protein sequence received by MALDI-TOF/TOF were manually added using the insertion tool implemented in the Discovery Studio suite (Accelrys, San Diego, CA, USA). Loop refinement was prepared by a multi-step CHARMm protocol implemented in Discovery Studio. The calculations are based on the ab initio loop prediction algorithm LOOPER. A set of low-energy conformations is generated for the specified loop region and scored based on their CHARMm energy [40].

The structures were visualized using the PyMOL Molecular Graphics System, Version 2.0, Schrödinger, LLC.

## 4. Conclusions

This study provides comprehensive insights into the kinetic properties, thermal stability, and structural characteristics of cysteine synthase A from *L. reuteri* LR1. The kinetic parameters were determined using two methods: the previously developed HILIC method [9] and the ninhydrin method, which is commonly employed for most cysteine synthases. A comparative analysis of the data obtained showed that the results of measurements of the catalytic parameters for o-acetylserine are similar. A new method for determining the activity of cysteine synthase can be used for similar enzymes. Structural analysis of LreCysK and its detailed comparison with structures from other organisms revealed that traditionally isolated regions of the amino acid sequence may not be sufficient for a comprehensive structure-functional analysis. Therefore, based on the obtained results, we assume that CysK enzymes have previously uncharacterized amino acid regions that are involved in the enzyme activity. LreCysK’s stability and function in varying environmental conditions make it a promising candidate for medical and industrial applications.

LreCysK’s unique properties, such as high thermal stability and catalytic activity, make it a promising candidate for biotechnological applications. In the industry, it can be used as a producer of cysteine [41]. We think that in the food industry, the enzyme could be incorporated into probiotic formulations to enhance the nutritional and health benefits of fermented products. Its ability to function efficiently in the human gut suggests that it could help to study the mechanism of cysteine and other sulfur-containing compounds production in detail. These compounds are essential for maintaining redox balance and cellular health [26]. This study may help develop new, more efficient methods for producing these compounds. Additionally, the enzyme can serve as a target for antibiotics and as an antimicrobial agent itself [8,42].

Future studies should investigate the engineering of LreCysK to enhance its activity or stability, thereby fully realizing its biotechnological potential. Also, this research on LreCysK could focus on several key areas, including the development of small-molecule inhibitors or activators that could modulate its activity. We assume that these compounds could be useful in studying the enzyme’s role in cellular metabolism or in developing new therapeutic strategies targeting sulfur metabolism in pathogenic bacteria. Additionally, exploring the role of LreCysK in the gut microbiome, particularly its interactions with other microbial species and its impact on host health, could provide valuable insights into the broader implications of cysteine metabolism in the gut.

## Figures and Tables

**Table 1:** Kinetic parameters of CysK enzymes. Parameters for the enzymes obtained in this work are in bold.

Enzyme	K_M_^OAS^, mM	K_M_^Sulfide^, mM	*k_cat_*, s^−1^	*kcat*/K_M_^OAS^ s^−1^mM^−1^	*kcat*/K_M_^Sulfide^ s^−1^mM^−1^	Source
LreCysK (HILIC)	3.8 ± 0.6	ND	681 ± 45	179	ND	This work
LreCysK (Ninhydrin)	3.05 ± 0.8	ND	1665 ± 167	546	ND	This work
*Salmonella typhimurium* LT-2 CysK	1.0 ± 0.6	0.006 ± 0.003	130 ± 17	130	21,667	[13]
*Lactobacillus casei* FAM18110 CysK	0.6 ± 0.1	6.7 ± 0.3	ND	ND	ND	[14]
*Leishmania donovani* CysK	15.86 ± 1.68	0.17 ± 0.01	ND	ND	ND	[15]
*Leishmania major* CysK	17.5 *±* 4.8	0.13 ± 0.04	ND	ND	ND	[16]
*Trichomonas vaginalis* G3 CysK	39.5 ± 2.9	0.8 ± 0.3	153 ± 44	3.9	191	[17]
*Aeropyrum pernix* K1 CysK	21	0.3	156	7.4	520	[18]
*Arabidopsis thaliana* CysK	1.4 ± 0.2	0.22 ± 0.9	1780 ± 280	1271	8090	[19]
*Escherichia coli* NK3 CysK	4.8	0.5	2030	422	4060	[20]
*Leucaena leucocephala* CysK	0.84 ± 0.03	0.06 ± 0.003	72.83	86.7	1213	[21]
*Leishmania infantum* CysK	3.5	ND	10.8	3.1	ND	[7]
*Trypanosoma theileri* CysK	2.4	ND	5.5	2.3	ND	[7]
*Trypanosoma cruzi* CysK	3.1	ND	4.8	1.6	ND	[7]

ND—no data.

**Table 2:** The enthalpy of denaturation and melting temperatures of domains of CysK apo and holo forms.

Domain	Tm, °C	ΔH, kJ/Mol
CysK domain_1	72.3	460
CysK domain_2	83.5	108.2
CysK + PLP domain_1	80.1	45.1
CysK + PLP domain_2	88.9	735.1

**Table 3:** Structural comparison of CysK enzymes deposited in the PDB database. Abbreviations used below are shown in brackets. Superposition of amino acid sequences was made with CysK from *L. reuteri* LR1. * Current paper.

Organism Type	Organism	PDB Code	Resolution, Å	RMSD	Space Group	Sequence Identity, %	Percentage of the Aligned Residues, %
Bacteria	*L. reuteri*	9JXUapo	2.2	-	P 1 21 1	-	-
	LR1(LreCysK*apo*) *						
Bacteria	*S. typhimurium*	6Z4Nholo	1.2	2.91	P 21 21 21	49	98
	(StyCysK)						
Bacteria	*Mycobacterium*	2Q3Cholo	2.1	2.68	P 41 21 2	55	98
	*tuberculosis*						
	(MtuCysK)						
Bacteria	*Plactomyces*	5XOQholo	1.87	2.89	P 2 21 21	54	97
	*limnophilus*						
	(PliCysK)						
Bacteria	*M. ulcerans*	4I1Yapo	2.6	2.03	P 1	55	96
	(MulCysK)						
Bacteria	*G. kaustophilus*	2EGUapo	1.9	2.33	P 43 21 2	66	97
	(GkaCysK)						
Protozoa	*Entamoeba*	4JBNholo	1.9	2.92	P 41	43	91
	*histolytica*						
	(EhiCysK)						
Protozoa	*L. donovani*	3SPXapo	1.79	2.82	P 21 21 2	49	97
	(LdoCysK)						
Protozoa	*T. cruzi*	8B9Yholo	1.8	2.42	P 1 21 1	45	100
	(TcrCysK)						
Plants	*A. thaliana*	1Z7Wholo	2.2	2.56	P 43 21 2	53	97
	(AthCysK)						

**Table 4:** Data collection and refinement statistics.

Parameter	Data
Diffraction source	Rigaku OD XtaLAB Synergy–S
Wavelength (Å)	1.54
Temperature (K)	100
Detector	HyPix–6000HE
Crystal–to–detector distance (mm)	34
Rotation range per image (*◦*)	0.5
Total rotation range (*◦*)	260
Space group	P2_1_
a, b, c (Å)	53.64, 101.29, 63.53
*α*, *β*, *γ* (*◦*)	90.00, 107.33, 90.00
Average mosaicity (*◦*)	0.93
Resolution range (Å)	20.40–2.20 (2.27–2.20)
Completeness (%)	98.9 (98.1)
Average redundancy	4.8 (5.0)
(I/*σ*(I))	5.4 (1.3)
Rmeas (%)	31.6 (149.6)
CC_1/2_	97.3 (41.2)
R_work_ (%)	21.2
R_free_ (%)	27.1
RMSD Bonds (Å)	0.02
RMSD Angles (*◦*)	2.94
Ramachandran favored (%)	96.1
Ramachandran allowed (%)	3
Ramachandran outlier’s region (%)	0.9
PDB entry code	9JXU

## Data Availability

The original contributions presented in this study are included in the article/Supplementary Materials. Further inquiries can be directed to the corresponding author.

## Supplementary Materials

The following supporting information can be downloaded at: https://www.mdpi.com/article/10.3390/ijms27010327/s1.

## References

[1] Kharwar S., Bhattacharjee S., Mishra A.K. (2021). Bioinformatics analysis of enzymes involved in cysteine biosynthesis: First evidence for the formation of cysteine synthase complex in cyanobacteria. 3 Biotech.

[2] Singh R.P., Saini N., Sharma G., Rahisuddin R., Patel M., Kaushik A., Kumaran S. (2021). Moonlighting Biochemistry of Cysteine Synthase: A Species-specific Global Regulator. J. Mol. Biol..

[3] Singh P., Brooks J.F., Ray V.A., Mandel M.J., Visick K.L. (2015). CysK Plays a Role in Biofilm Formation and Colonization by *Vibrio fischeri*. Appl. Environ. Microbiol..

[4] Benoni R., Beck C.M., Garza-Sánchez F., Bettati S., Mozzarelli A., Hayes C.S., Campanini B. (2017). Activation of an anti-bacterial toxin by the biosynthetic enzyme CysK: Mechanism of binding, interaction specificity and competition with cysteine synthase. Sci. Rep..

[5] Shaposhnikov L.A., Pometun A.A., Tishkov V.I. (2024). Lactobacilli and Klebsiella: Two Opposites in the Fight for Human Health. Biochemistry.

[6] Abuqwider J., Altamimi M., Mauriello G. (2022). Limosilactobacillus reuteri in Health and Disease. Microoganisms.

[7] Sowerby K., Freitag-Pohl S., Murillo A.M., Silber A.M., Pohl E. (2023). Cysteine synthase: Multiple structures of a key enzyme in cysteine synthesis and a potential drug target for Chagas disease and leishmaniasis. Acta Crystallogr. D Struct. Biol..

[8] Savinova O.S., Glazunova O.A., Moiseenko K.V., Begunova A.V., Rozhkova I.V., Fedorova T.V. (2021). Exoproteome analysis of antagonistic interactions between the probiotic bacteria *Limosilactobacillus reuteri* LR1 and *Lacticaseibacillus rhamnosus* F and multidrug resistant strain of *Klebsiella pneumonia*. Int. J. Mol. Sci..

[9] Ma W., Wang J., Li Y., Wang X. (2019). Cysteine synthase A overexpression in *Corynebacterium glutamicum* enhances l-isoleucine production. Biotechnol. Appl. Biochem..

[10] Wyres K.L., Nguyen T.N.T., Lam M.M.C., Judd L.M., Chau N.V., Dance D.A.B., Ip M., Karkey A., Ling C.L., Miliya T. (2020). Genomic surveillance for hypervirulence and multi-drug resistance in invasive *Klebsiella pneumoniae* from South and Southeast Asia. Genome Med..

[11] Joshi P., Gupta A., Gupta V. (2019). Insights into multifaceted activitiesof CysK for therapeutic interventions. 3 Biotech.

[12] Chernobrovkina A.V., Gorbovskaia A.V., Chikurova N.Y., Les E.K., Efremova A.D., Chichkanova E.S., Shpigun O.A., Tishkov V.I., Pometun A.A. (2026). Development of hydrophilic interaction liquid chromatography method for determining enzymatic activity of cysteine synthase A. J. Chromatogr. A.

[13] Tai C.H., Nalabolu S.R., Jacobson T.M., Minter D.E., Cook P.F. (1993). Kinetic mechanisms of the A and B isozymes of O-acetylserine sulfhydrylase from *Salmonella typhimurium* LT-2 using the natural and alternate reactants. Biochemistry.

[14] Bogicevic B., Berthoud H., Portmann R., Meile L., Irmler S. (2012). CysK from *Lactobacillus casei* encodes a protein with O-acetylserine sulfhydrylase and cysteine desulfurization activity. Appl. Microbiol. Biotechnol..

[15] Singh K., Singh K.P., Equbal A., Suman S.S., Zaidi A., Garg G., Pandey K., Das P., Ali V. (2016). Interaction between cysteine synthase and serine O-acetyltransferase proteins and their stage specific expression in *Leishmania donovani*. Biochimie.

[16] Williams R.A.M., Westrop G.D., Coombs G.H. (2009). Two pathways for cysteine biosynthesis in *Leishmania major*. Biochem. J..

[17] Westrop G.D., Goodall G., Mottram J.C., Coombs G.H. (2006). Cysteine Biosynthesis in *Trichomonas vaginalis* Involves Cysteine Synthase Utilizing O-Phosphoserine. J. Biol. Chem..

[18] Mino K., Ishikawa K. (2003). Characterization of a Novel Thermostable O-Acetylserine Sulfhydrylase from *Aeropyrum pernix* K1. J. Bacteriol..

[19] Bonner E.R., Cahoon R.E., Knapke S.M., Jez J.M. (2005). Molecular Basis of Cysteine Biosynthesis in Plants: Structural and functional analysis of O-acetylserine sulfhydrylase from *Arabidopsis thaliana*. J. Biol. Chem..

[20] Mino K., Yamanoue T., Sakiyama T., Eisaki N., Matsuyama A., Nakanishi K. (2000). Effects of Bienzyme Complex Formation of Cysteine Synthetase from *Escherichia coli* on Some Properties and Kinetics. Biosci. Biotechnol. Biochem..

[21] Harun-Ur-Rashid M., Oogai S., Parvee S., Inafuk M., Iwasak H., Fukut M., Amzad Hossai M., Oku H. (2019). Molecular cloning of putative chloroplastic cysteine synthase in Leucaena leucocephala. J. Plant. Res..

[22] Hunter J.E., Harper A.E. (1976). Stability of some pyridoxal phosphate-dependent enzymes in vitamin B-6 deficient rats. J. Nutr..

[23] Montioli R., Zamparelli C., Voltattorni C.B., Cellini B. (2017). Oligomeric State and Thermal Stability of Apo- and Holo- Human Ornithine δ-Aminotransferase. Protein J..

[24] Chattopadhyay A., Meier M., Ivaninskii S., Burkhard P., Speroni F., Campanini B., Bettati S., Mozzarelli A., Rabeh W.M., Li L. (2007). Structure, Mechanism, and Conformational Dynamics of O- Acetylserine Sulfhydrylase from *Salmonella typhimurium*: Comparison of A and B Isozymes. Biochemistry.

[25] Ågren D., Schnell R., Oehlmann W., Singh M., Schneider G.C. (2008). Synthase, (CysM) of *Mycobacterium tuberculosis* Is an O-Phosphoserine Sulfhydrylase. J. Biol. Chem..

[26] Campanini B., Benoni R., Bettati S., Beck C.M., Hayes C.S., Mozzarelli A. (2015). Moonlighting O-acetylserine sulfhydrylase: New functions of an old protein. Biochim. Biophys. Acta.

[27] Rabeh W.M., Cook P.F. (2004). Structure and mechanism of O-acetylserine sulfhydrylase. J. Biol. Chem..

[28] Les E.K., Pometun E.V., Savin S.S., Tishkov V.I., Pometun A.A. (2025). Cysteine synthase: A key enzyme of cysteine synthetic pathway. Biochem. (Mosc.).

[29] Shaposhnikov L.A., Chikurova N.Y., Atroshenko D.L., Savin S.S., Kleymenov S.Y., Chernobrovkina A.V., Pometun E.V., Minyaev M.E., Matyuta I.O., Hushpulian D.M. (2024). Structure–Functional Examination of Novel Ribonucleoside Hydrolase C (RihC) from *Limosilactobacillus reuteri* LR1. Int. J. Mol. Sci..

[30] Koshkina M.K., Shelomov M.D., Pometun A.A., Savin S.S., Tishkov V.I., Atroshenko D.L. (2023). Speeding up SDS-PAGE: Theory and experiment. Electrophoresis.

[31] Chikurova N.Y., Gorbovskaia A.V., Stavrianidi A.N., Fedorova E.S., Shemyakina A.O., Buryak A.K., Uzhel A.S., Chernobrovkina A.V., Shpigun O.A. (2023). Novel Adsorbents for the Determination of Amino Acids in Soil Extracts by Hydrophilic Interaction Liquid Chromatography with Mass Spectrometric Detection. J. Anal. Chem..

[32] Shaposhnikov L.A., Chikurova N.Y., Chernobrovkina A.V., Tishkov V.I., Pometun A.A. (2024). Development of an Approach to Determining Enzymatic Activity of Ribonucleoside Hydrolase C Using Hydrophilic Interaction Liquid Chromatography. J. Chromatogr. A.

[33] Markov D.I., Zubov E.O., Nikolaeva O.P., Kurganov B.I., Levitsky D.I. (2010). Thermal Denaturation and Aggregation of Myosin Subfragment 1 Isoforms with Different Essential Light Chains. Int. J. Mol. Sci..

[34] Abramson J., Adler J., Dunger J., Evans R., Green T., Pritzel A., Ronneberger O., Willmore L., Ballard A.J., Bambrick J. (2024). Accurate structure prediction of biomolecular interactions with AlphaFold 3. Nature.

[35] Vagin A.A., Isupov M.N. (2001). Spherically averaged phased translation function and its application to the search for molecules and fragments in electron-density maps. Acta Crystallogr. D Biol. Crystallogr..

[36] Winn M.D., Ballard C.C., Cowtan K.D., Dodson E.J., Emsley P., Evans P.R., Keegan R.M., Krissinel E.B., Leslie A.G.W., Mccoy A. (2011). Overview of the CCP4 suite and current developments. Acta Crystallogr. D Biol. Crystallogr..

[37] Emsley P., Lohkamp B., Scott W.G., Cowtan K. (2010). Features and development of Coot. Acta Crystallogr. D Biol. Crystallogr..

[38] Krissinel E., Henrick K. (2004). Secondary-structure matching (SSM), a new tool for fast protein structure alignment in three dimensions. Acta Crystallogr. D Biol. Crystallogr..

[39] Krissinel E., Henrick K. (2007). Inference of Macromolecular Assemblies from Crystalline State. J. Mol. Biol..

[40] Spassov V.Z., Flook P.K., Yan L. (2008). LOOPER: A molecular mechanics-based algorithm for protein loop prediction. Protein. Eng. Des. Sel..

[41] Caballero Cerbon D.A., Gebhard L., Dokuyucu R., Ertl T., Härtl S., Mazhar A., Weuster-Botz D. (2024). Challenges and Advances in the Bioproduction of L-Cysteine. Molecules.

[42] Kaushik A., Rahisuddin R., Saini N., Singh R.P., Kaur R., Koul S., Kumaran S. (2021). Molecular mechanism of selective substrate engagement and inhibitor disengagement of cysteine synthase. J. Biol. Chem..

