# Supplementary material for: Structure-Functional Examination of Cysteine Synthase A (CysK) from Limosilactobacillus reuteri LR1
Source: Int J Mol Sci. 2025 Dec 28;27(1):327. doi: 10.3390/ijms27010327 (PMC12785664; doi:10.3390/ijms27010327)
Supplement: Supplementary file 1 [file ijms-27-00327-s001.zip › ijms-4013301-supplementary.pdf]

Figure S1. SDS PAGE for LreCysK

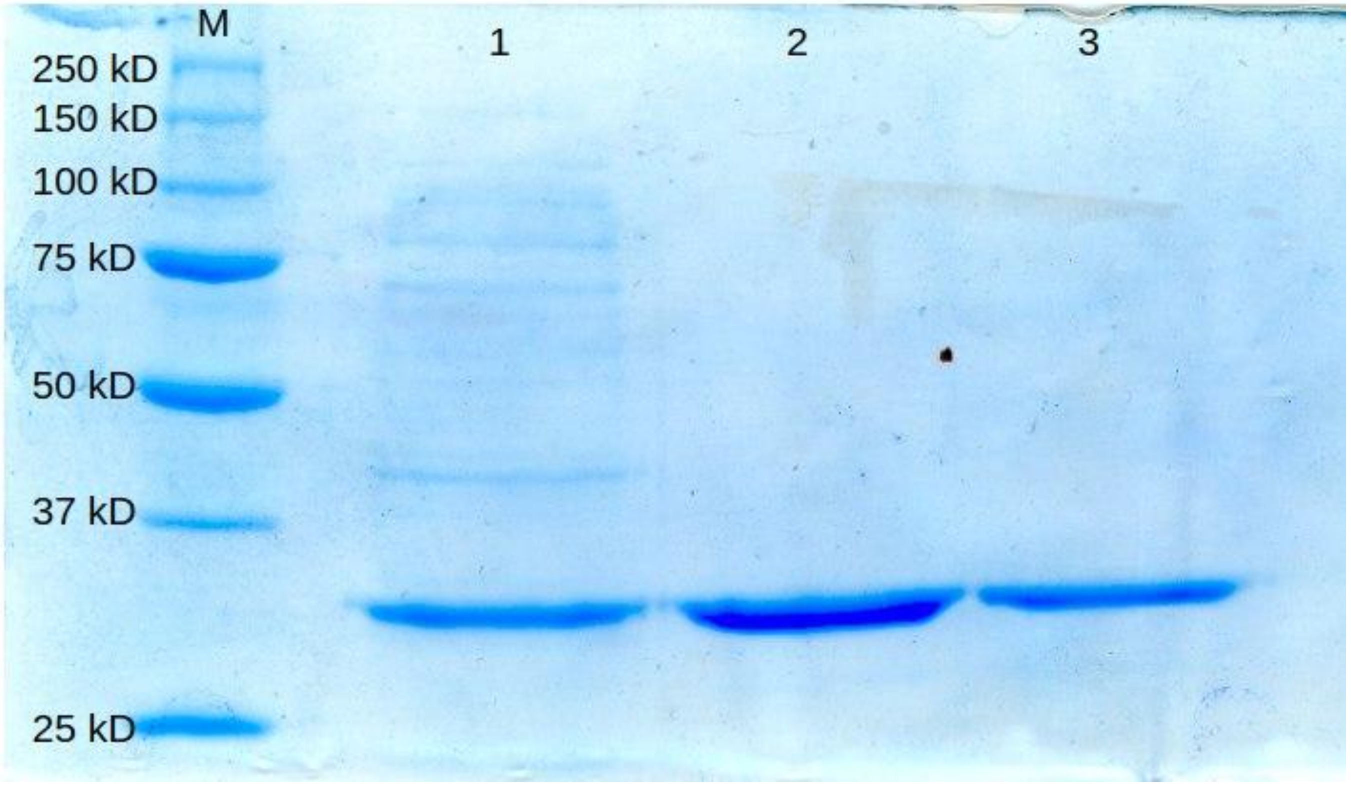

M – molecular weight markers, 1 – LreCysK extract after sonification, 2 – LreCysK solution after metal chelate chromatography, 3 – LreCysK solution after desalting

Figure S2 MALDI analysis with the theoretical sequence of LreCysK

Protein sequence coverage: 91%

Matched peptides shown in ***bold red***.

```
  1 MHHHHHHTKI TNSIVNLIGN TPIVKLNRVV PEDAADVYVK LEFFNPGGSI
 51 KDRIALAMIE EAEKAGKLQA GGTIVEPTSG NTGVGLAMVA AAKGYHLVIT
101 MPETMSVERR KLMQGYGAEL ILTPGADGMK GAIAKAEELV KEKGYFMPMQ
151 FDNPANPAIH EETTGKEILE AFGDDIPDAF VAGVGTGGTL TGVGHALKKA
201 NPNVQIYALE PAESPVLKEG KGGKHKIQGI SAGFIPKVLD TDVYNGILEI
251 KSDDAITMAR EVGHQEGILV GISAGANIKG AIEVAKKLGK GKQVITVAPD
301 GGDRYLSTEL FNY
```

Table S1: Calculated parameters for determination of LreCysK oligomeric state.

Protein	Kav	lgMr
Lactalbumin	0.5253	4.15
Ovalbumin	0.3797	4.64
Conalbumin	0.2911	4.87
Aldolase	0.1392	5.20
Ferritin	0.0759	5.64
Thyroglobulin	0.0127	5.83
LreCysK	0.3418	4.74

Figure S3. Chromatogram for calibration curve using size-exclusion chromatography

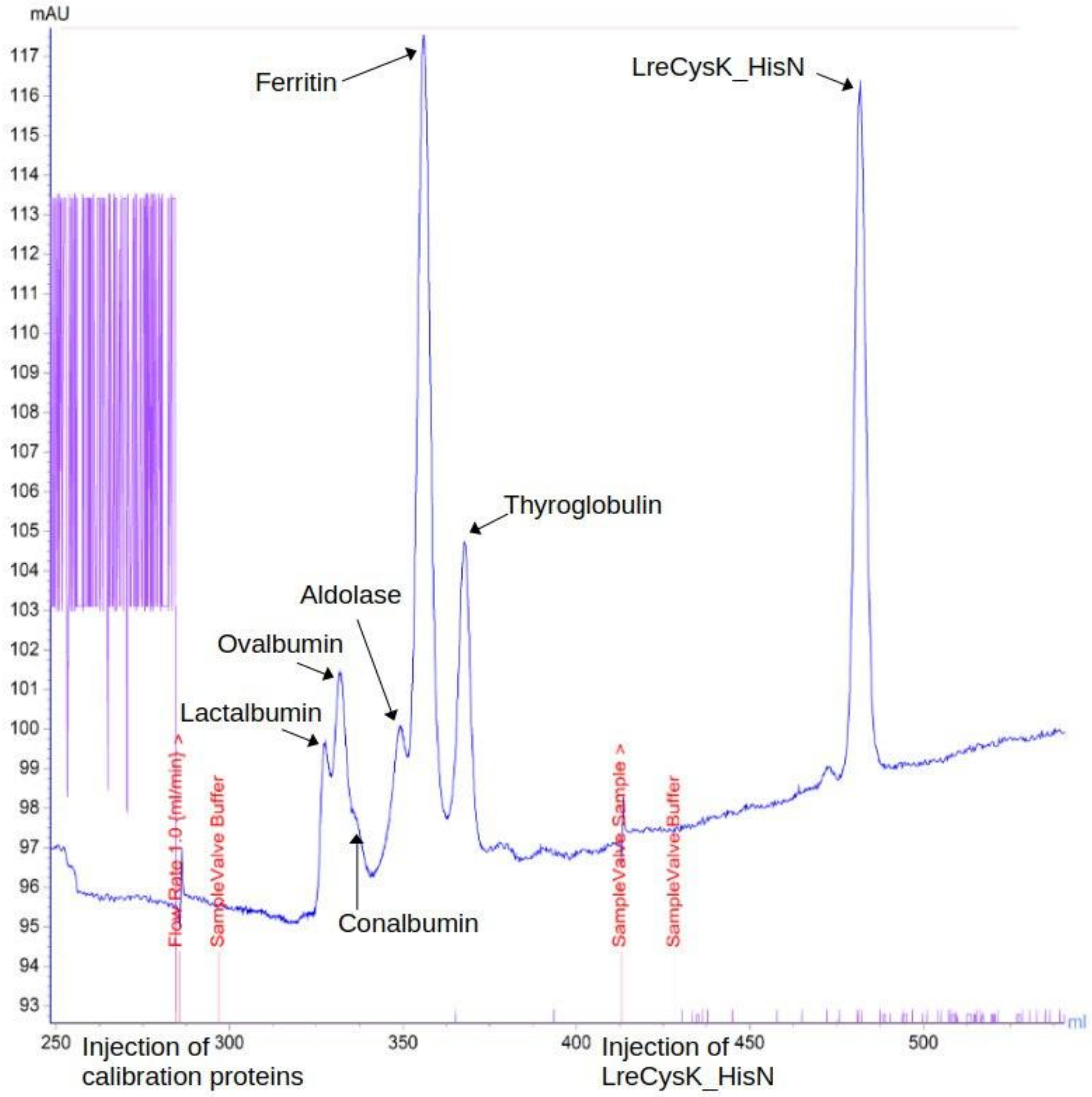

S4. Determination of K_M^{OAS} for LreCysK

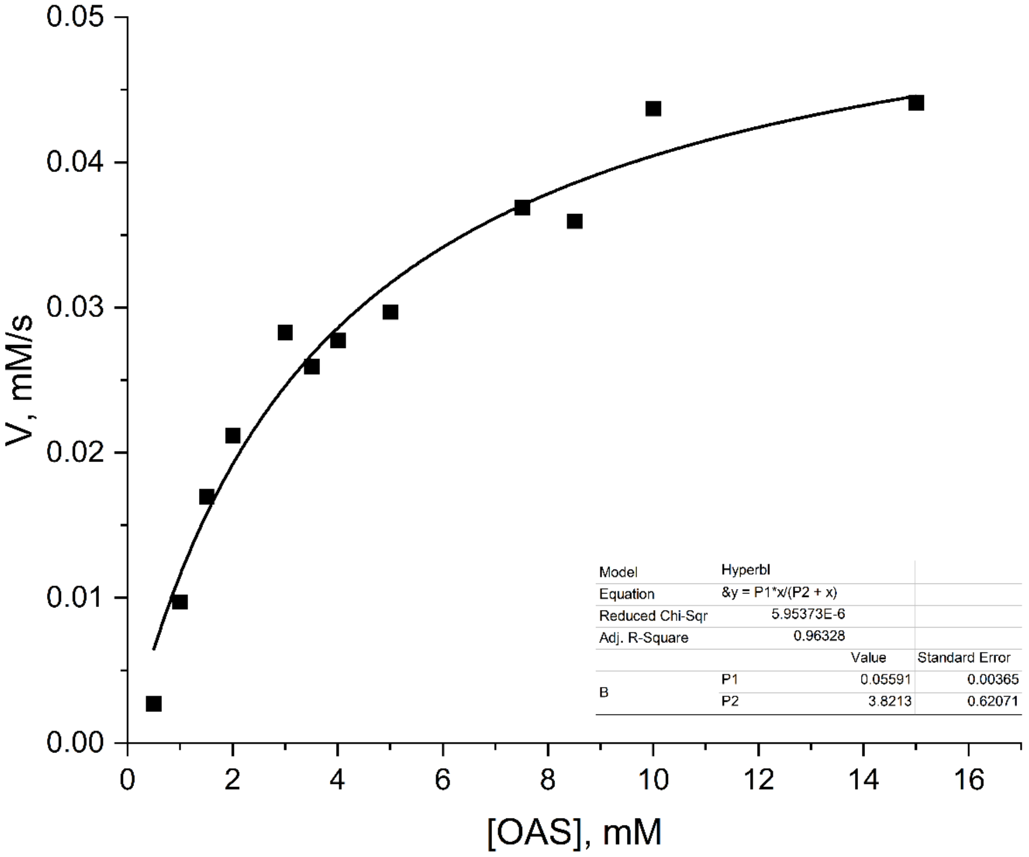

HILIC method, CysK concentration 2.9 mkg·mL^{-1}

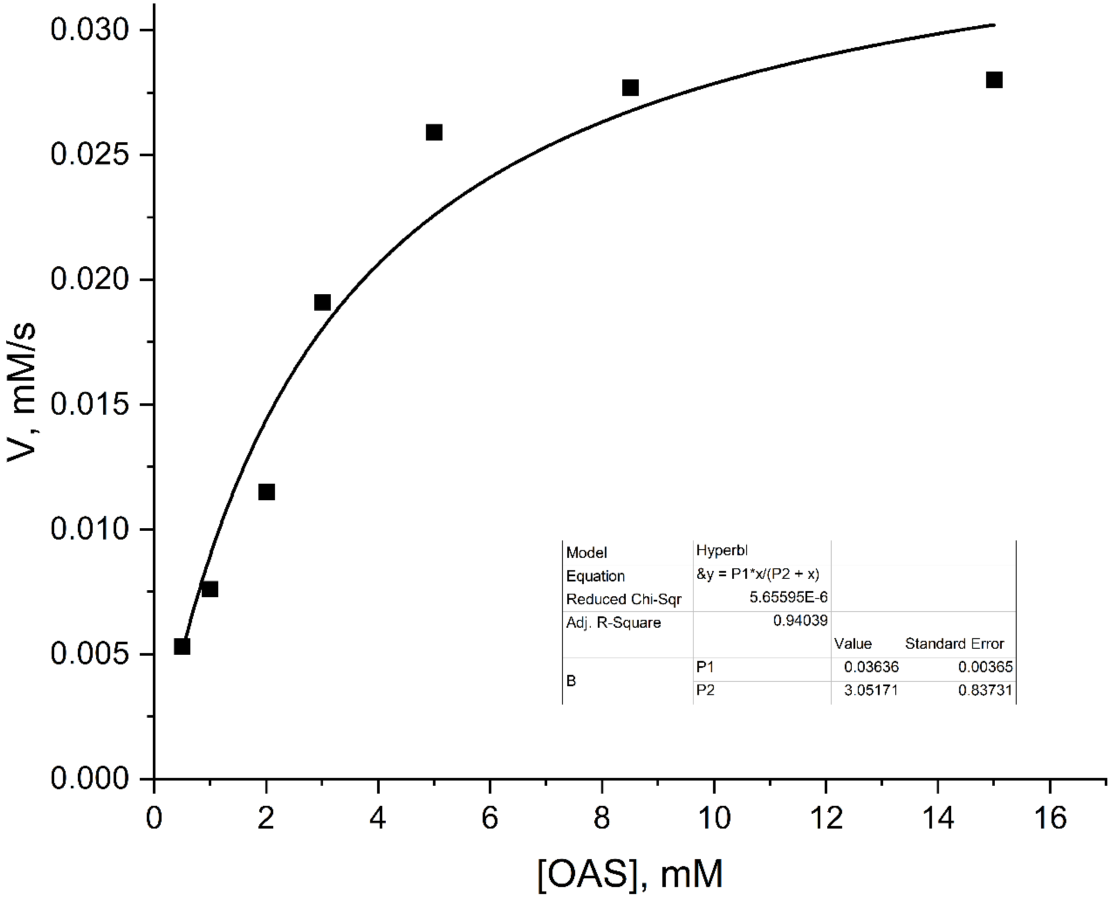

Ninhydrine method, CysK concentration 0.77 mkg·mL^{-1}

S5 Phylogenetic tree of CysK with studied kinetic parameters

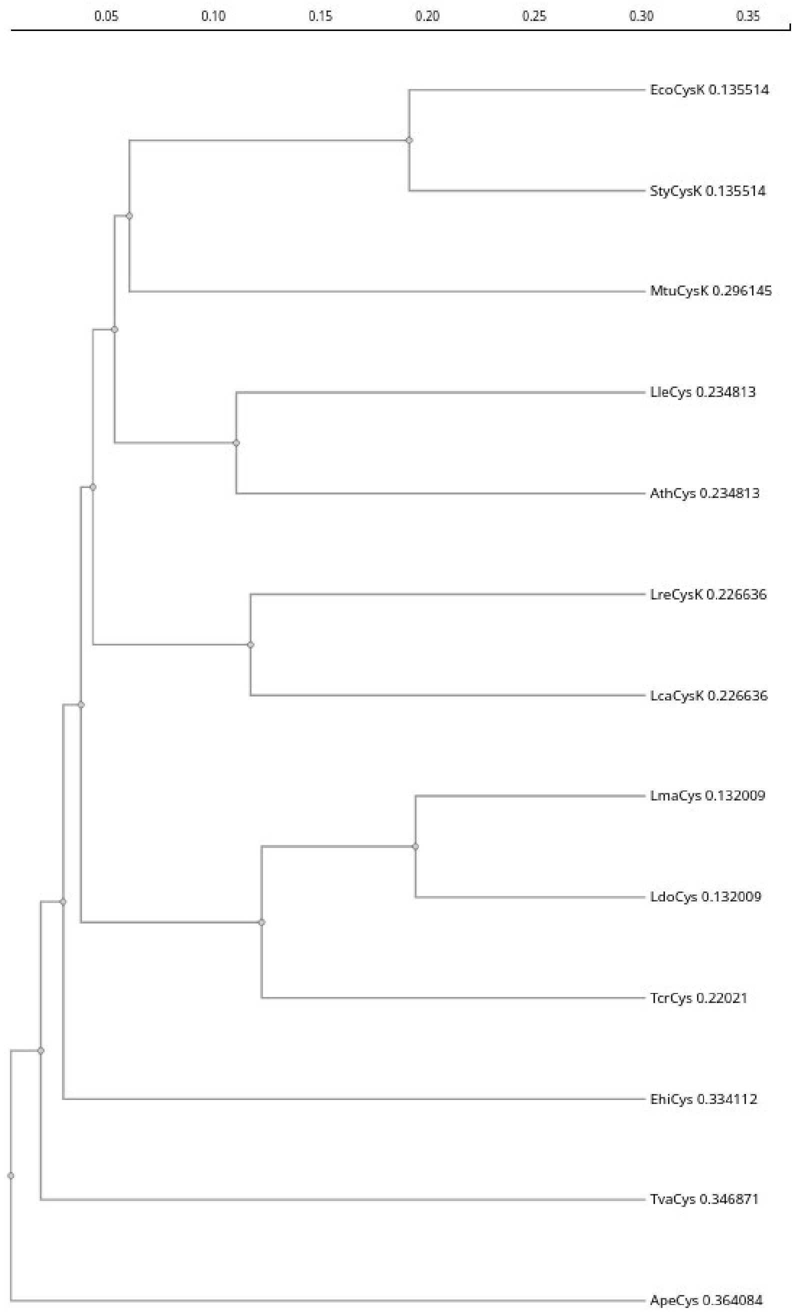

S6. Multiple sequence alignment of CysK from different lactobacilli.

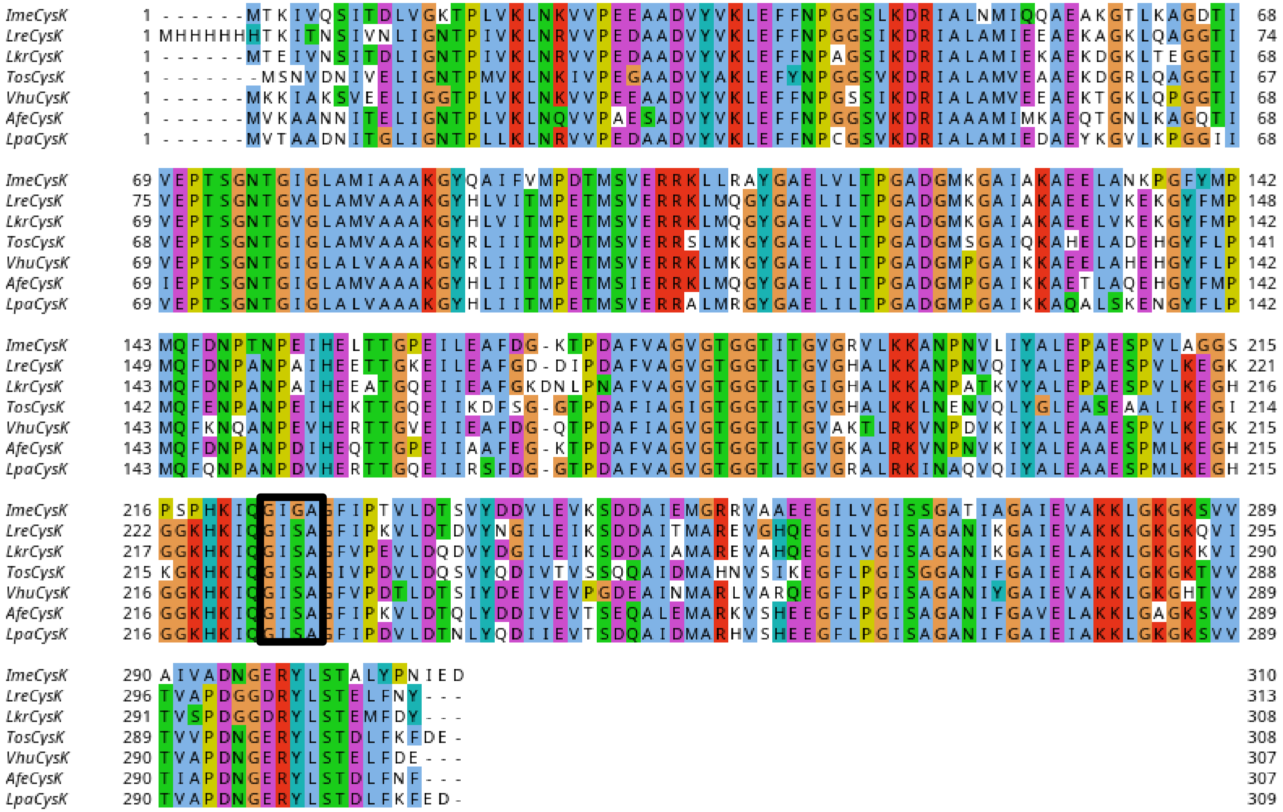

ImeCysK – *Isobaculum melis* (NCBI Reference Sequence: WP_092652743.1), LreCysK – CysK from *L.reuteri* (GenBank: MBU5982312.1), LkrCysK – *Limosilactobacillus kribbianus* (NCBI Reference Sequence: WP_267202021.1), TosCysK – *Tetragenococcus osmophilus* (NCBI Reference Sequence: WP_123935669.1), VhuCysK – *Vagococcus humatus* (NCBI Reference Sequence: WP_125943125.1), AfeCysK – *Agrilactobacillus fermenti* (NCBI Reference Sequence: WP_230913953.1), LpaCysK – *Lacticaseibacillus paracasei* (NCBI Reference Sequence: WP_123019069.1). GIGA/GISA loop are highlighted in box.
